# Partial Nephrectomy for T1b/T2 Renal Mass: An Added Shift from Radical Nephrectomy

**DOI:** 10.15586/jkcvhl.v9i4.255

**Published:** 2022-10-06

**Authors:** Mohamed Sharafeldeen, Wael Sameh, Vahid Mehrnoush, Amer Alaref, Radu Rozenberg, Asmaa Ismail, Hazem Elmansy, Walid Shahrour, Ahmed Zakaria, Osama Elmeslemany, Nishigandha Burute, Anatoly Shuster, Owen Prowse, Ahmed Kotb

**Affiliations:** 1 Department of Urology, Faculty of Medicine, Alexandria University, Alexandria, Egypt.; 2 Department of Urology, Northern Ontario School of Medicine, Thunder Bay, Ontario, Canada

**Keywords:** eGFR, large renal mass, nephrometry score, partial nephrectomy, radical nephrectomy

## Abstract

The aim of our study was to show our short-term experience in managing large renal masses (cT1b/T2) through partial nephrectomy (PN) over the last 3 years. Retrospective data collection for all patients managed by PN for renal masses larger than 4 cm over the last 3 years. Epidemiological data were collected. Surgical data including surgical and ischemic times as well as intra and postoperative complications were collected. Pre- and postoperative estimated glomerular filtration rate (eGFR) data were collected and correlated as well as postoperative complications and recurrence. We could identify 47 patients managed by PN for radiologically confirmed >4 cm renal masses. The mean age of the patients was 55.7 ± 13.4, including 29 males and 18 females. Masses were T1b and T2 in 40 and 7 patients, respectively. The mean tumor size was 6.2 ± 1.5 cm. Using renal nephrometry score; 8, 28, and 11 had low, moderate, and high complexity, respectively. Renal cell carcinoma (RCC) was identified in 42 patients. Five patients out of 42 cancerous cases (12%) had pathological T3 RCC. The mean preoperative and postoperative eGFR were 89.09 ± 12.41 and 88.50 ± 10.50, respectively (P 0.2). The median follow-up was 14 months and within that short time, no patient had evidence for cancer recurrence. PN for large renal masses is safe in experienced hands and should be attempted in a higher percentage of patients, regardless of the tumor complexity. No cancer recurrence or deterioration of renal function was observed within our short-term follow-up.

## Introduction

Renal cell carcinoma (RCC) is becoming more common as imaging technology improves and the population ages (1). Both partial nephrectomy (PN) and radical nephrectomy (RN) are effective treatment options for patients with localized renal masses (cT1–T2). However, because of the added benefit of long-term renal function preservation, PN is the preferred surgical treatment for the majority of patients with cT1a renal masses (2, 3). The role of PN in the treatment of larger tumors (cT1b and cT2) is still being investigated (4). Previous reports for large renal mass revealed mixed results in terms of oncological outcomes and technological success (5, 6). While some studies have shown that PN can be performed safely in large tumors with acceptable technical, oncological, and functional outcomes (5, 7, 8), others have found no difference in survival between PN and RN patients (6). The purpose of this study was to demonstrate our short-term experience in managing large renal masses (cT1b/T2) via PN over the last 3 years.

## Methods

### Clinical data collection and follow-up

Retrospective data collection of all patients treated by PN for renal masses larger than 4 cm over the last 3 years. Demographic, pathologic, operative, and postoperative data were obtained from the medical records. Postoperative complications were evaluated and classified using the Clavien–Dindo grading system for surgical complications (9).

### Statistical analysis

Patient age, tumor size, estimated glomerular filtration rate (eGFR), and ischemia time are all presented as mean ± standard deviations. Other information, such as sex, complexity score, type of tumor, pathological results, and complications are expressed as numbers and percentages.

### Consent and ethical approval

Patients’ consent for data collection and publishing was obtained. Institutional ethical approval was obtained.

## Results

We were able to identify 47 patients who were being treated by two surgeons by PN for radiologically confirmed >4 cm renal masses. The patients’ mean age was 55.7 + 13.4, with 29 males and 18 females. In 30 and 17 patients, the tumor was found on the right and left sides, respectively. There were 44 patients (93.6%) with solid enhancing mass and three patients with Bosniak 3/4 renal cysts. T1b and T2 masses were found on radiographs in 40 and 7 patients, respectively. The mean tumor size was 6.2 + 1.5 cm (Range: 4.5–10.5 cm). Renal nephrometry scores of 8 (17%), 28 (60%), and 11 (23%) indicated low, moderate, and high complexity, respectively. All cases were managed through open surgery, with 45 and 2 being treated via retroperitoneal and transperitoneal approaches, respectively.

Warm and cold ischemia were used to treat 40 and 7 cases, respectively. The median warm and cold ischemic times were 10 and 25 min, respectively. There was no need for a blood transfusion in any of the cases. The drain was removed on the first postoperative day, and all patients were discharged 48–72 h later. One patient (2%) developed a Grade 3 complication as he returned to the emergency department 1 week after surgery with hypotension and low hemoglobin. A CT angiogram revealed a bleeding pseudoaneurysm that was successfully managed by the interventional radiology team.

**Figure 1:**
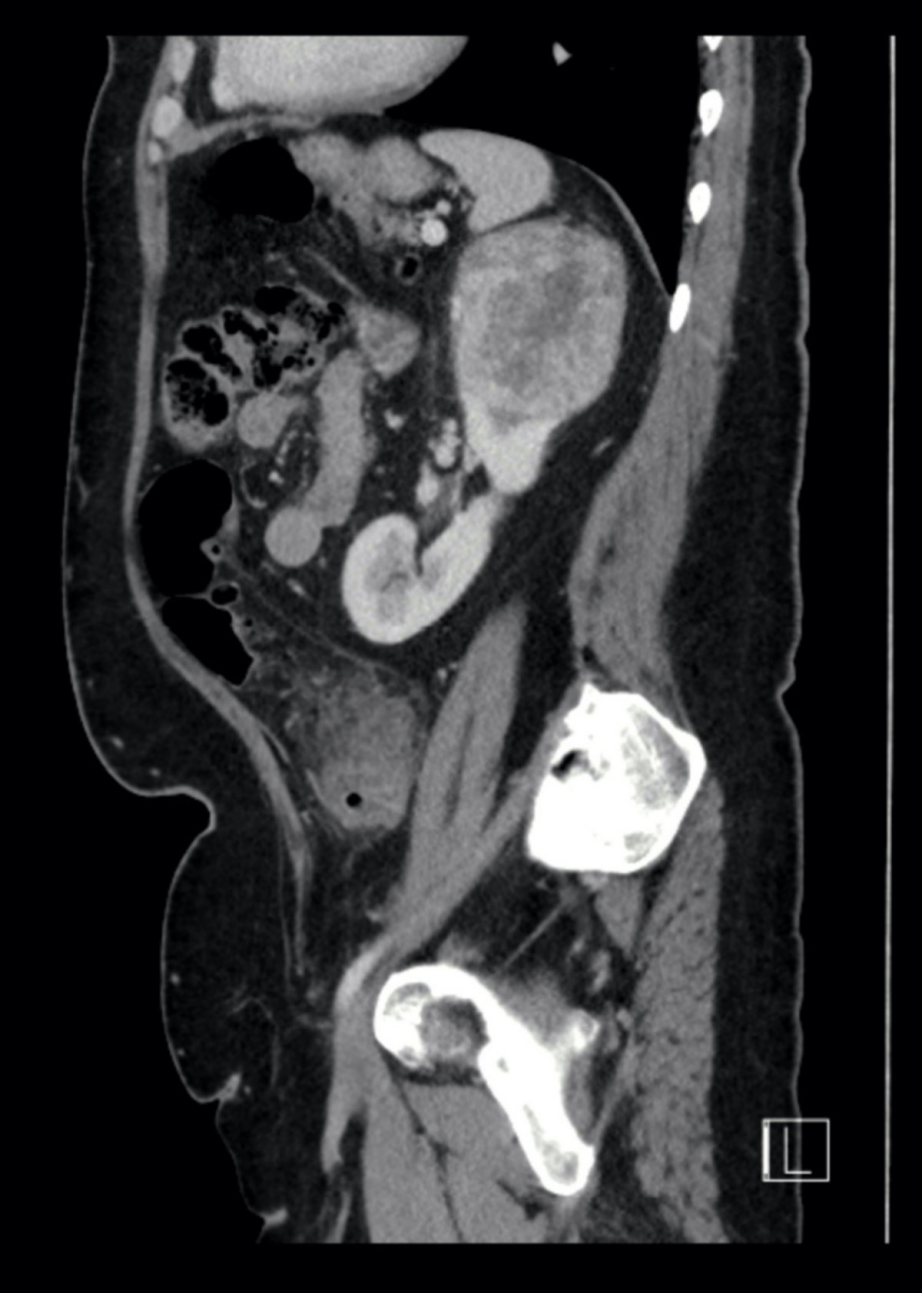
Sagittal CT view showing upper pole right renal tumor.

**Figure 2:**
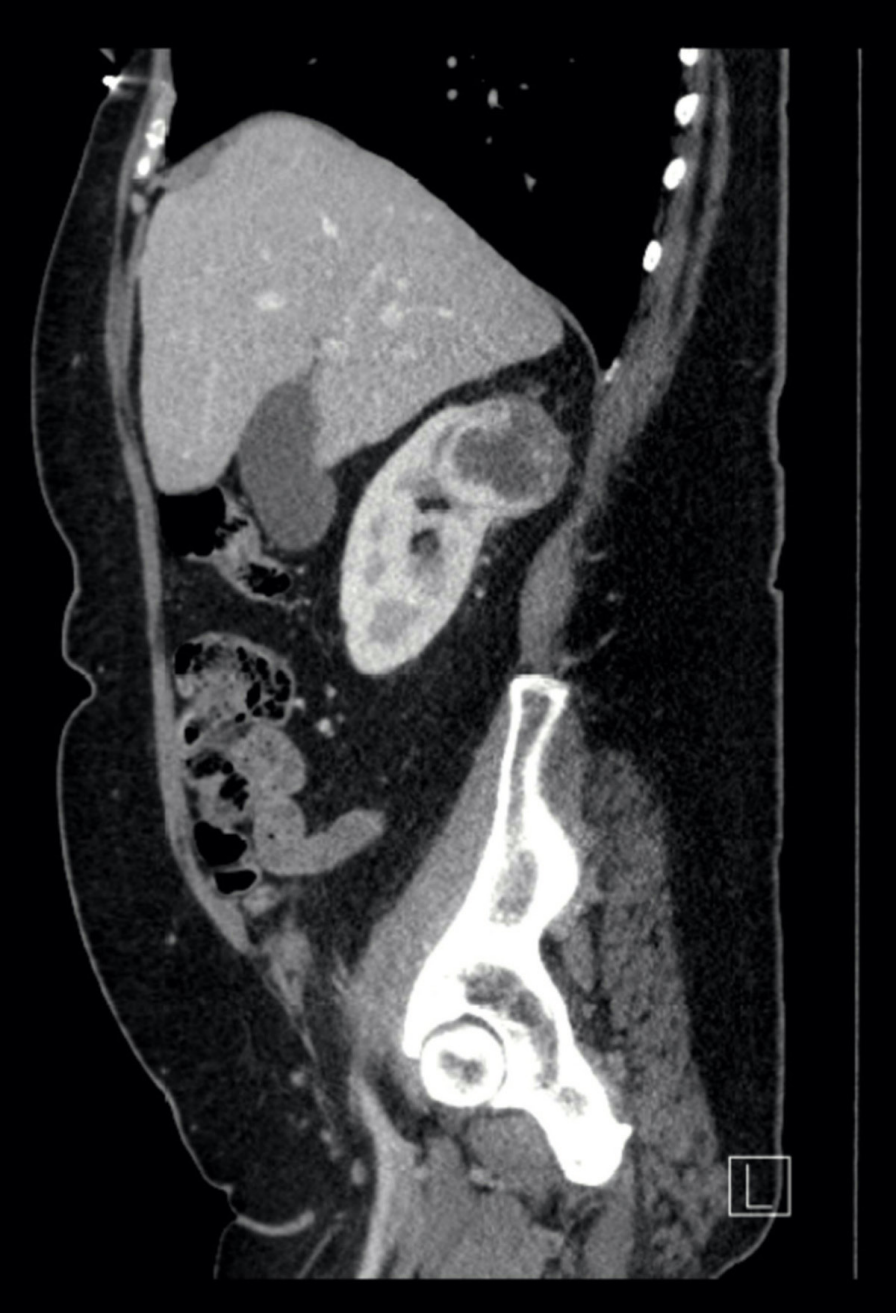
Sagittal CT view showing upper pole right renal tumour.

**Figure 3:**
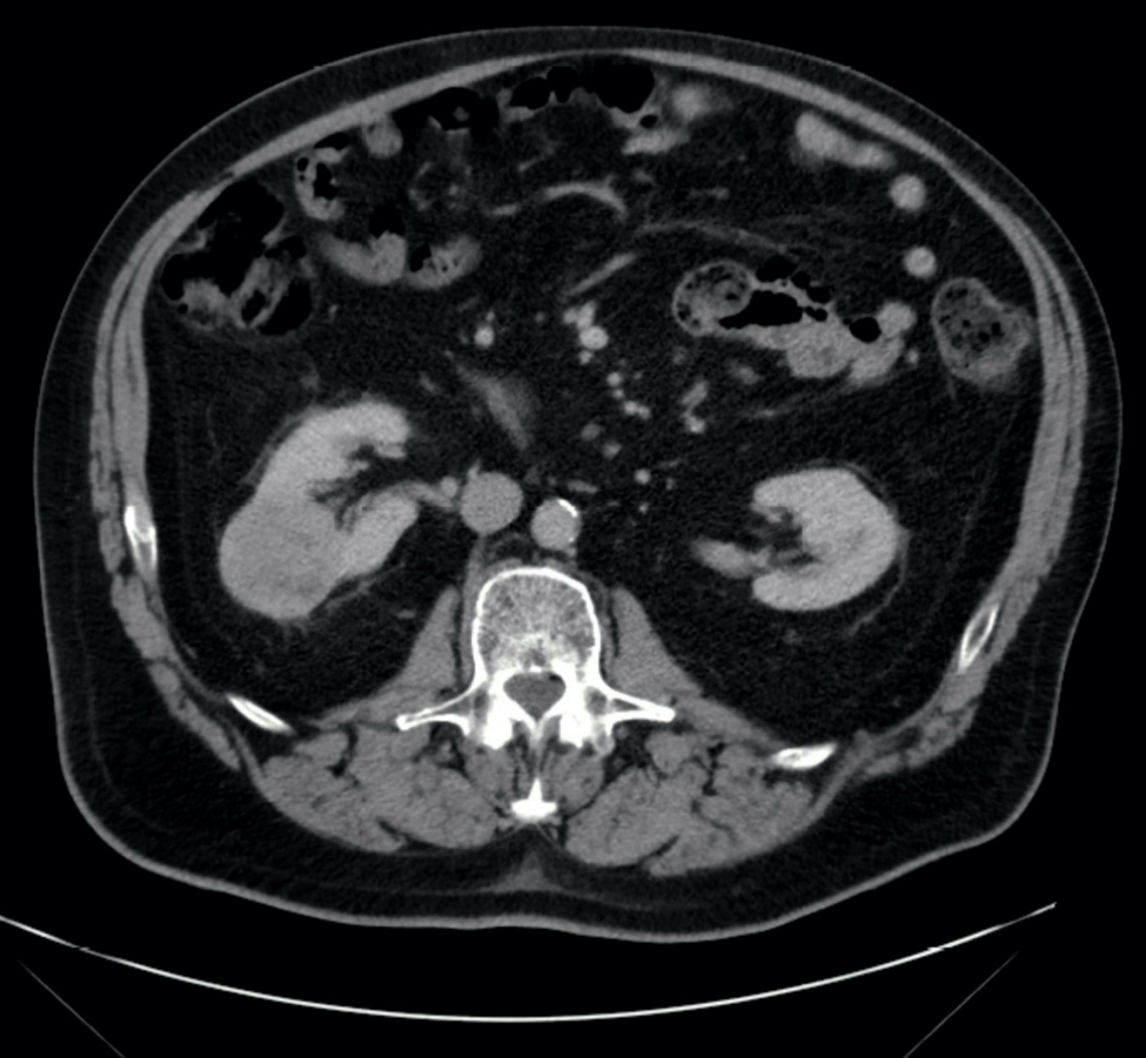
Axial CT view showing right renal mass.

RCC was found in 42 patients, while oncocytoma and low-fat angiomyolipoma were found in five others. Negative surgical margins were found in 45 patients (96%), while positive surgical margins were found in two patients (4%). Five patients (12%) out of 42 cancerous cases had pathological T3 RCC, and none had a positive margin. The preoperative and postoperative eGFR were 89.09 ± 12.41 and 88.50 ± 10.50, respectively (P = 0.2). The median follow-up was 14 months, and no patient had evidence of cancer recurrence during that time. Figures 1–3 show some of our cases. Table 1 illustrates the clinical and pathological outcomes of the studied patients.

**Table 1:** Clinical and pathological outcomes.

Variable			Outcomes
Age (years)	Mean ± SD		55.7 ± 13.4
	Range		37–80
Sex (n)	Males		29
	Females		18
Laterality (n)	Right		30
	Left		17
Tumor size (cm)	Mean ± SD		6.2 ± 1.5
	Range		4.5–10.5
Clinical stage (n)	T1b		40
	T2		7
Tumor characteristic (n)	Solid		44
	Complex cystic		3
R.E.N.A.L. nephrometry score (n)	Low complexity		8
	Moderate complexity		28
	High complexity		11
Ischemic type and time	Warm	n	40
		Median (min)	10
	Cold	n	7
		Median (min)	25
Pathology	RCC		42
	Benign		5
Margins	Negative		45
	Positive		2
Pathological stage	T1b/T2		42
	T3		5

## Discussion

We retrospectively summarized the medical records of 47 patients with large renal masses managed by PN. According to our findings, the size of the renal masses ranged from 4.5 to 10.5 cm, with 60% having moderate complexity and 23% having high complexity. Based on radiographic evaluation, 85.1% were T1b, and 4.9% were T2. Pathological findings revealed that 89.4% of patients had RCC, with the remainder having oncocytoma or low-fat angiomyolipoma. Regarding the recurrence rate, no patient had evidence of cancer recurrence during the follow-up period.

Our experience with open PN has yielded promising results for treating large renal masses, with only 2% of grade 3 complications necessitating further management reported during postoperative evaluation and patient follow-up. In terms of complications, some studies did show that PN was associated with a higher complications rate compared to RN (4, 10, 11). In a study by Long et al., 16 complications with a median (range) follow-up of 13.1 months, including 8.2% blood transfusions and 12.2% urinary fistulae, were reported from 46 cases of large renal tumor managed by PN (5). According to a recent meta-analysis of 13 retrospective studies including 2906 patients (PN: 1172; RN: 1734) comparing the efficacy and safety of PN versus RN, PN was linked to a longer operative time and higher estimated blood loss. PN had a significantly higher risk of low-grade and high-grade surgical complications compared to RN (11).

Despite the fact that PN has more complications than RN, PN has a higher overall survival rate. PN, as opposed to RN, preserves renal parenchyma and protects against renal function deterioration caused by noncancer causes (12). Some population-based studies supported the PN and RN cancer-specific mortality equivalence for T1aN0M0 and T1bN0M0 RCC. As a result, in such patients, PN should be given equal consideration to RN based on cancer control equivalence (7, 8). In patients with small RCC less than 4 cm, PN has shown equivalent oncologic outcomes to RN, while preserving renal function and possibly increasing survival (2, 3). In tumors larger than 4 cm, PN was safe and yielded positive results (13).

PN has been shown to benefit patients with T2 renal tumors in terms of survival and renal function protection (14). Overall and RCC-specific 5- and 10-year survival rates after PN have been reported to be 94.5 and 70.9%, respectively (5). In a meta-analysis of 21 case-control studies involving 11,204 patients (RN 8620; PN 2584), cancer-specific mortality was lower for PN (4). However, some studies found no differences in survival rate between PN and RN. The result of one study comparing survival after PN versus RN among 11,256 cases of RCCs of 4–7 cm between 1998 and 2007 found no difference in survival in patients after adjusting for tumor size and age (6). A recent retrospective review of patients who underwent PN (n = 72) or RN (n = 379) for cT2 renal masses from 2000 to 2016 also found that metastases-free and cancer-specific survival were not significantly different between groups in patients with RCC (10).

There were no differences in eGFR between the preoperative and postoperative periods, according to our findings. This is consistent with previous findings that the decrease in eGFR after PN is significantly lower than that after RN (10, 11). A meta-analysis included 15 retrospective studies involving 5056 patients who had nephrectomy (PN: 1975, RN: 3081). The decline in eGFR after PN was less than that after RN. Subsequently, PN has been shown to benefit patients with T2 renal tumors in terms of renal function protection (14).

Although several studies have demonstrated that PN, whether performed as open surgery or as a laparoscopic procedure, has a satisfactory outcome (5, 7, 8, 15), results of a meta-analysis of 13 studies involving 13,269 patients (PN = 6145 and RN = 7124) found that the clinical efficacy of RN was superior to that of PN to some extent; however, PN had a faster recovery and was a less harmful therapy. Based on their findings, the operating time, glomerular filtration rate, and recurrence were significantly different between PN and RN (16).

A major limitation of the study is that it is a retrospective study looking at patients managed with PN. This actually is expected to miss patients that were managed with RN following a failed attempt with PN. While our study confirms the safety and applicability of PN in the case of large renal masses, it does not point to the actual success rate to fulfill that target.

## Conclusion

PN for large renal masses is safe in experienced hands and should be attempted in a higher percentage of patients, regardless of the tumor complexity. No cancer recurrence or significant deterioration of renal function was observed within short-term follow-up. In consequence, PN should be attempted whenever technically feasible.
